# Commentaries on *the Organon**Contributed to the proceedings of The International Hahnemannian Association at its meeting in June, 1898.

**Published:** 1898-08

**Authors:** B. Fincke

**Affiliations:** Brooklyn, N. Y.


### COMMENTARIES ON *THE ORGANON*.*

(The Problem of Potentiation, *Organon*, Section 916, 280 Note Sq.)

B. FINCKE, M. D., BROOKLYN, N. Y.

### POTENTIATION *versus* EVOLUTION.

Hahnemann in no matter has been more felicitous in expression than when he called the force-developments *Kraftentwickelungen*) of medicine potencies (*Potenzen*), an expression which recommends itself at once in two aspects. Very soon, in his peculiar method of developing the latent medicinal forces of substances by means of a large quantity of an inert vehicle, he found that there was no thinkable test to show the developed force aside from the physiological one furnished by the organism in its reaction upon it. Driven, therefore, to acknowledge in the medicine prepared by distributing it in a large quantity of vehicle in a certain constant ratio, a power to act upon the living organism of man, he could no better express the nature of these preparations than by the term *Potency*—*i. e.*, a power to act if its energy is called into requisition by the homœopathic law. Furthermore, adopting the ratio of 1 : 100 for his proportion of medicine to vehicle, the mathematical conception of involution, viz., raising a number to a potency desired came in quite naturally for application in homœopathic therapeutics, inasmuch as the root of the potency was one drop of fluid medicine-substance mixed with 99 drops of an inert fluid vehicle under suitable succusion, or one grain of dry medicine triturated with 99 grains of an inert vehicle, by which he obtained the first centesimal potency. This answered the purpose of notation very well, because the exponents would indicate in every instance how many times the crude substance had been distributed

---

* Contributed to the proceedings of The International Hahnemannian Association at its meeting in June, 1898.

throughout the vehicle in the centesimal ratio. However, this seems to be not quite correct, because at every new potency the preparation acquires new properties of medicinal action, which militates against the mathematical idea of a "power," which is a product developed from *equal* factors, for the different potencies of a medicine-substance are not equal factors, because their value by virtue of their potential quality is determined only by the organism upon which it is to act, according to homœopathic law.

However, if we admit that the magnitude which is subjected to involution is also constant in the homœopathic potentiation, since the remedy, in spite of its variability of action in its potencies, remains the same—Sulphur always is Sulphur in crude substance or in highest potency—the adaptation of the mathematical conception of involution for the homœopathic pharmacy is quite correct, and proves a happy discovery of the old master. The term potency is to be preferred to all other terms already, for the reason that in its variable quantity of mixtion with inert vehicles it not only denotes the diminution of the substance and its refinement and final disappearance, but also the invariable quality of the organic action without losing its firm practical standpoint, which awards to it the power to act on the organism, though it cannot be tested by physico-chemical methods alone. The term potency, therefore, is to be preferred to all other terms.

Thus the term "dilution" is inappropriate, because it relates only to the distribution of the medicine-substance to the fluid vehicle, which soon comes to an end when the limit of materiality is reached and is inadequate to express the development and continuation of the medicinal force contained in the crude substance beyond the limit of materiality. Dilutions, properly speaking, can only refer to objects of taste and color, says Hahnemann, and can never be dynamizations (*Chronic Diseases*, Vol. V, preface).

Attenuation is a term which can only find its appropriate use as far as the materiality of the substance is concerned, and is likewise unfit for the higher potencies. These remain true attenuations or dilutions, but no dynamizations.

Dynamization is a term frequently used by Hahnemann, expressing exactly the same meaning as potency, and depends already upon the doctrine of Leibnitz that every substance involves force, which certainly is verified by Hahnemann's doctrine of potentiation. It has at the same time the advantage of being derived from the Greek *dynamis,* which has already been acknowledged in the doctrine of mechanics in physics, under the name of dynamics, and lies at the foundation of the whole Hahnemannian doctrine of homœopathics. It is then a matter of taste to use the term potency or dynamization, and relieves the monotony of expression of one and the same conception. Still the term potency recommends itself for its being brief and practical in denoting the power of medicine to act on the living organism.

In Section 16 of his *Organon* (fifth ed.), Hahnemann gives us in his comprehensive manner the instruction that all diseases originate in the dynamic change of the living organism, and that it can only be removed and therefore healed by similar dynamic remedies, the preparation of which he later on describes. This, he says emphatically, is the science and art of healing, termed by him appropriately homœopathics.

The postulatum for preparing powerful remedies, therefore, would be that they are to be developed from the crude substances so as to exert in their administration the necessary dynamic character similar to that of disease, but no material action whatever. Now the discovery of potentiation by Hahnemann was so out of the common and went so glaringly against the natural laws propounded by physics and chemics, which in their development assumed to fathom the origin, essence, and being of all things that his precious gift to mankind has been ridiculed, despised, and rejected as useless by most physicians, natural philosophers, and other wise men, nay, even by the great majority of adherents to the homœopathic profession. They have raised the banner *Similia Similibus* unknowingly as antagonistic to the doctrine of potentiation, and going against the very safest formation of homœopathics. They have, some purposely and some unwittingly, overlooked that Hahnemann persists upon the immateriality and dynamicity of disease, which to him is nothing more than a spirit-like, dynamic, virtual change of health in the system, a change in that something which keeps the body alive, regulating and arranging in it the contending and antagonistic or assimilative physical and chemical forces. This something he calls vital force, life-force, and without it no individual can live. It is by the wonderful natural potentiation that the body is built up after the creation and incarnation of the spirit, and develops to maturity, and then is preserved in more or less integrity and constant activity till death ensues. Some physiologists assert that matter forms the spirit by the inherent eternal properties of matter, and they have built a great castle in the air, which tumbles down with the death of every man. No; spirit forms the organism from the minutules of matter, and its force out of the mysterious, unfathomable depth of God. "*A Jove principium.*" "In the beginning was the Word, and the Word was with God, and the Word was God. The same was in the beginning with God. All things were made by Him, and without Him was not anything made that was made. In Him was life, and the life was the light of man." But it is not necessary here to enter into a controversy about religion.

Suffice it to say that Hahnemann admits of no other source of disease than a noxious dynamic change in the life-force. shown by the symptoms subjective and objective, and of no other remedy than what has been obtained from substances through appropriate preparation and potentiation in order to acquire the dynamic, spirit-like character to render it similar to the life-force, and thus being able to meet the enemy successfully.

It appears, therefore, to be inconsistent when Hahnemann thought the physiological quality of his so-called infinitesimal potencies were to be developed by friction and succussion, because these modes of motion could not be expected to do more than to free the minutular particles of the crude substance from their contact and cohesion with their fellows, and to use an unmedicinal vehicle for distributing them through it.

A mystical change of the vehicle by the mechanical action of succussion and trituration was not to be expected, and has indeed been rejected of right by the materialists of our school. It was also not admissible because Hahnemann himself wanted not this faulty conception, but the liberation and development of the dynamic spirit-like nature of the substance, its medicinal force, similar to the spirit-like life-force of the organism. This, therefore, is not admissible, and what has been said about it, especially in the preface on Dilutions and Potencies in the last volume of his *Chronic Diseases* must be disowned and rejected in the light which the development of the so-called high potencies since the last sixty years has shed upon this subject, and also as inconsistent with his own fundamental principles so well expressed in the same preface, when he says: "Homœopathic *dynamizations* are true awakenings of the medicinal properties latent in the natural bodies during their crude state, which then are enabled to act almost spiritually upon our life—*i. e.*, upon our sentient (sensible) and irritable fibre." But in facilitating the potentiation in the beginning of the process the mechanical rubbing and shaking may be of use for certain substances and advisable to a limited extent. Who can say that the drop of medicinal juice which has gone through a series of vials, each filled with 99 drops of alcohol, by dropping over one drop of the previous vial into the succeeding one even only thirty times, as Hahnemann did, actually has been diluted or attenuated thirty times on the ratio of one to a hundred? The relation of drug and vehicle is neither that of dilution nor of attenuation, because on account of the short limit of materiality we have no means to prove it. Hahnemann, himself, says very truly that real dilutions only occur with objects of taste and color, and so also attenuations can only be made of solid objects, such as wire, minerals, powders, etc. For these reasons the terms dilution and attenuation are deemed inappropriate. Hence in the light of potentiation the dilution and attenuation must indeed have taken place in the lower stages, but they are more incidental, and though of necessary occurrence in assisting mechanically the liberation and consequent distribution of the medicinal forces to the inert vehicles applied, not the *conditio sine qua non* for the development of the higher potencies. There is a short limit to attenuation of solids or liquids which the spectroscope assigns to the ninth or tenth centesimal potency. It is, therefore, by no means to be admitted that because a substance has been detected spectroscopically in the ninth or tenth centesimal potency, its dilution or attenuation would continue to the 100,000th potency and higher, for the conception of matter ceases at such dizzy heights, and even at much lower degrees as even a Hahnemannian 30th centesimal. It is just the same with attenuation of solids, which comes soon enough to an end, as the microscopists of our own school have shown who assign the limit to lay between the twelfth and thirteenth centesimal, and the fine platina wire that Wollaston drew out is not to be compared with a thirtieth centesimal potency at all. Jaeger's and Fincke's Neural Analysis does not prove any more. It is the physiological test shown by suitable electric apparatus upon the sensitive living organism, but it does not prove that the medicine-substance is present in a state of dilution or attenuation in the potencies examined.

It only proves that there is something in the potency which acts and acts more or less powerfully in the human body. If Hahnemann says somewhere (Section 280, note) that the attenuation of the drug-medicine cannot go on forever, that there must always be something of the original substance left, he could not mean anything else than that the medicinal force of that drug was left, being conveyed to the highest potency by the transference of it upon the inert vehicle. Crookes' radiant matter stops at a limit of a third centesimal potency, and falls short of comparison with even a Hahnemannian thirtieth centesimal potency.

Some other terms expressing the incalculable distribution of a medicinal force through a great mass of inert vehicle might fall into the selection of a proper term—*e. g.*, rarefaction taken from the air, refinement from drawing out of fine wires, or trituration of powders, force-developments as exponents of mechanical force, dynamization from the Greek *dynamis*, or force, but potency will carry the palm, because it is all-sufficient for scientific and practical purposes, and expresses exactly what it means, the energy of medicinal force—*potentia et actu.*

Besides, in the investigations about the minuteness of things we always come at last to the discrepancy between the concreteness of things and expansion. It happens in this way: A mass of matter considered as an aggregation of minutules* (atoms, molecules) can artificially be divided up into

---

* The *infinitesimal* is a quantity belonging to a series of magnitudes running into infinity of minuteness, and is perfectly unassignable.

The *minutule* denotes a magnitude, or rather partitude, exceedingly small but still assignable.

The *atom* is the ultimate simple quantity of matter in chemics and physics, assignable.

The *molecule* is the ultimate compound quantity of matter assignable.

We, therefore, cannot borrow the molecular and atomic theory from the physico-chemical school, because referring to matter. The minutules referring to force suffice to indicate the limit of our high potencies, discrete particles, which every one forms a concrete again, liable to disgregation *ad infinitum.* But here the modification of method comes in. We may conceive a particle of a mass to be extremely minute (molecule) and yet under certain circumstances assume a greater magnitude by expansion—*e. g.*, by conversion into a gas, or by exhaustion through the air-pump. This is a seeming contradiction, but it is only seeming, because a new element appears in the process of diminution, viz., expansion which clearly makes the particle occupy more space than it had before. Now we can again, if we possess the means to do it, subdivide the minutest particle of this expansive mass of the particle. But who possesses the means? It does not go on *ad infinitum.* There are certain limits for every kind of matter, but we cannot assign them. Physical science is constantly busy to extend those limits of materiality, but without success. It is even so as with the astronomical explorations which with more powerful glasses discover new myriads of heavenly bodies without coming to an end.

There is, however, this difference: the chemical and physical processes by which simple substances are brought into such conditions that they present the character of increasing fineness, are very various and involve the most intricate knowledge of the chemical and physical laws. The vehicles there used, so to say, are not one simple substance, like sugar of milk, or alcohol, or water, but all kinds of substances with which they are mixed in order to be separated again by others and under various conditions of chemical affinity, heat, light, magnetism, electricity, motion, etc. But in our homœopathic potentiation the manipulation is most simple and its philoso-

---

which are assigned by their specific action upon the organism under the homœopathic law.

The infinitesimal and minutule refer to force, the atom and molecule to matter. The infinitesimal is unassignable except by thought. The minutule is assignable by its action upon the sensitive living organism.

phy easily understood. We need not go into the region of the unexplainable and be carried away by the fascination of occult sciences, religious speculations, most intricate mathematical computation, and sophistical artifices of reasoning, but keep down to the solid ground of matter of fact, which nobody can deny, when by practice and repetition the action of high potencies is proved again and again. In our case the simple, pure substance to be subjected to the process of potentiation is simply mixed with an inert vehicle in a regular proportion of a minute quantity of substance, say 1 to a large quantity of vehicle, say 99 in appropriate time, and ascending in a series of potencies, the exponent of which always indicates the renewed ratio of 1 : 100. In this manner we arrive at altitudes of diminution and refinement and rarefaction as regards the substance used, and contrariwise a magnitude of expansion and distribution of its medicinal force which surpasses everything thus far known in science.

The calculations designed to prove that compound substances cannot exceed a certain minuteness in size because at that stage its constituents would fall asunder in atoms, fails utterly here since the very fact of potentiation teaches that such compounds retain their specific character from the lowest to the highest potencies. They as such are individuals which no more change in their dynamic nature than simple substances, or plants, or animals, or men. They retain their individuality as long as their identity is not touched, but, of course, they have not the spiritual individuality of man, which survives even his death, because their individuality is only spirit-like, not spiritual.

Comparing with this view the molecular hypothesis, the latter falls short of the truth, because it assumes atoms as the simplest small elements of matter which by combination in virtue of the chemical affinity form compound smallest elements of matter from which the larger aggregations are obtained. It is assumed that the smallest molecule will always contain two atoms, and in its ultimate condition will divide into two atoms of the substances of which it is composed. But our high potencies show in their highest numbers the unchangeable character of the combination in their action upon the living organism. This discrepancy depends upon the circumstance that the medicinal forces as high potencies do not follow the chemical laws which govern matter, but the dynamical laws which govern life.

In our case the means of showing the substance in its highest potencies is the finest reagent which the world is able to furnish, viz., the human and animal organism in health and disease. The difficulty of observation upon such fine natural instruments of precision cannot be claimed as an excuse for not taking the subject of potentation into close scientific consideration. A good and true homœopathician can often tell from the data of his *Materia Medica Pura* which variations from health to disease may belong to the influence of the potency used, just as well as which variations from disease to health may likewise have to be owing to the influence of a high potency applied to the sick. We, therefore, cannot expect from a Crookes or Tyndall an investigation into the merits of homœopathic potentiation, as Dunham once thought, because it is not in their line, and we cannot likewise shirk the responsibility to show how the potentiation of substances is in necessary relation to physical and chemical processes, but extremely simple and typical and not subject to the arbitrary forms which some scientists have pleased to assign to matter according to physico-chemical laws. Their atoms are but a figment of the brain, and their molecules are still concrete masses subject to further diminution, which at the same time includes expansion, not otherwise to be obtained in the aggregate condition of matter.

Korsakoff made the ingenious experiment of placing one medicated globule of an already potentiated substance as high as 30th centesimally into a vial filled with unmedicinal globules, and subjecting the vial to succussion for a little while. Then the medicated globule was taken out again. When testing the previously unmedicinal globules it was found that they acted homœopathically in the same direction as the medicated globule, from which Korsakoff concluded that all the globules had been, as it were, infected by the medicinal one. The inference from this fact upon the origin of contagious diseases was evident to him immediately, and so he declared. Though the causes of this class of diseases are everywhere called poisons, nobody ever has been able to isolate and demonstrate them *ad oculos*, and therefore it is very justifiable on our part to assume that those hypothetical poisons are high potencies prepared in nature's own laboratory by a potentiation thus far unknown. To assign the origin of infectious and contagious diseases to the presence of bacteria and their supposed excretions is not justified, because bacteria found in diseased conditions and deemed characteristic for them, have also been found in healthy organisms without causing disease. They cannot do harm in the organism before the dynamic change in it from health to disease has taken place. They are, therefore, an example of the much reproved *post hoc*, but not *propter hoc*, and stand in the relation of parasites, which on the appropriate homœopathic treatment disappear without leaving any trace behind them.

But this medication by contact is apt to throw also some light upon the potentiation by mixtion or fluxion. Where we reflect upon how the communication of the medicinal force of one globule to many inert globules may proceed, we are forced to the conclusion that the medicinal force must emanate and radiate from the one globule in all directions, and either be deposited upon or taken up and absorbed by the many inert globules, and thus rendered homœopathically active. Or it may be that the atmospheric air in the vial which is being infected or contaminated by the atmosphere of the medicine globule, and the inert globules surrounded by the atmospheric air receive their medicinal quality by its influence. If now we compare the fluid vehicle receiving into its substance the medicinal force by fluxion, to the atmospheric air constituting the vehicle of potentiation by contact, the *simile* is apparent, for the *modus operandi* after all is the same, though the measurement of the air-vehicle is nearly impossible and certainly impracticable. The potency entering the vial whilst coming in contact with the vehicle, spreads out in all directions and imparts itself to the minutules of the fluid contained therein. The difference is only in that this action continues uninterruptedly by fluxion in one single vial while with a contact-potency for every stated quantity of globules fresh vials have to be used. There is still this to be remarked, that the unmedicated globule taken out again has not lost any of its strength, and can be used again, and that the process of contact-potentiation can be continued from the first contact-potency onward. This shows a richness of nature in the medicinal forces which is truly inexhaustible, and is analogous to the richness of the original substances from which innumerable potencies can be derived without materially changing their nature at all—*e. g.*, in Aurum, Argentum, Carbo, Graphites, Platina, Sepia, Silicea, Adamas, etc. This infection itself rests upon the science of mechanics in the transference of force which we see in every motion whatever, large or small, in innumerable forms, so that the very familiarity with its results blinds people in regard to this indispensable principle of motion. Every wagon in the street (vehicle), every machine turning out work, every physical and mental exertion of man and animal conveys to thinking man the necessity of adopting this principle. If, therefore, there are medicinal forces in matter they must partake of the same phenomenon, that they can be transferred upon other matter and by the same principle. when administered to the healthy or sick organism, act in their specific manner by producing symptoms of disease on the healthy and removing symptoms of disease in the sick, all according to homœopathic law.

After these and similar considerations it is clear that we cannot expect from physical science the solution of the problem of potentiation. Nay, this wonderful process brings the question of all science at once home. Is there any spirit at all? Or is spirit only a function of matter? If not, of what nature is it, and whence is it? These questions are hoary with age, and have divided the mass of intelligent men into two great contending parties. There are those who assign to matter peculiar properties which have been from eternity and continue to eternity. From these properties all the rest flows in a Darwinian flux from the original speck of protoplasm at the bottom of the sea, that famous creature of Huxley's brain, the Bathybius, to the highest development in man, which all disappears after death like a phantasmagoria of a *lanterna magica,* and nothing is left behind but the unmerciful, immutable, eternal properties of matter which entertain the flux into eternity. To this comes the suggestion of the greatest mathematician of our age, who attributes the origin of man to minute cells derived from meteors, which are carried along in space for millions and millions of years, and preserved by the immense degrees of cold similar to those lately produced in a lesser degree by eminent chemists. These cells in the supposed meteors fell at an opportune time upon the earth, which nursed them as a kind of mother to produce the organic bodies, and in the course of other untold millions of years finally the human organism by evolution.

It is inconceivable how a divine can advocate evolution as taught by modern science, because it leads him in the opposite direction from the road he intends to travel, for evolution leads to agnosticism (a new term for scepticism), and atheism, which when pressed acknowledges an abstraction in the place of God. When Sir John Franklin traveled south to escape the Arctic terrors, he did not know that he was on an immense ice-field drifting incessantly northward, and carrying him to unavoidable perdition. These divines resemble him.

This evolution is the gist of the doctrine of the present representatives of science, and there are now in the old world where the beautiful egg has been hatched (and, alas! in the new one) not many natural and other philosophers who do not adore this modern idol of man, a mere abstraction, a hypothesis which leaves humanity to the merciless properties of matter. Fortunately all men must inevitably die at one or another period of their lives, or else the pride of scientific men would acquire a power which might be detrimental to the very existence of the human kind on earth. Fortunately all the popes of religious, scientific, and political persuasions, who hold themselves infallible and use their power to suppress what does not tally with their ideas and desires, must step down from their high stations when their allotted time comes. For science itself rectifies itself and purifies itself from the shortcomings of its votaries, its confessors and professors. Hypotheses which have served a temporary purpose of propping up weak parts in science, must give way when these parts are masoned up with solid work in unison with the plan of the whole building, for science is the great receptacle of human wisdom following creation, but cannot itself create.

But the Hahnemannian potentiation is not a theory, a hypothesis, but a solid fact which apparently helps to sustain the foundation of science inasmuch as Jaeger expresses it as thereby the limits of divisibility of matter are very much extended. This, however, cannot be accepted according to the light given by the higher and highest potencies which even this pioneer of science has not had an opportunity to prove; rather it should be said that it helps to understand better the relation of force and matter, how they react upon each other during life, how they cannot be separated without changing essentially the one or the other. Thus the spirit molds matter by means of its forces after conception into the independent organism which now lives its individual life, and in a most wonderful and intricate potentiation goes on to maturity which the material part of the body generally reaches sooner than the spiritual one until matter finally supervenes, and, no longer controlled by the life-force, drives the spirit out of his temporal abode, thus affording him the entrance into that other life which surely is to come after death. The relation of matter and force is strikingly illustrated in the custom of burial based upon the native religion of the Indians. Mr. McLean in his book on *The Indians,* says, page 31: "They believe that everything in nature is possessed of a spirit, and that the spirits of the articles devoted to the deceased depart with him and are used in the spirit-world. Thus when you point to the goods lying at the grave after many days, the natives will tell you that the substance remains, but the spirits live on the spirits of the things. The souls of the hatchets and pipes, horses and dogs go to the happy hunting grounds for their master's uses. We find the same idea of object-souls amongst the Arabs and Gauls. The camel of the Arab was tied near the grave of his master that he might there perish and follow him in the spirit-world." This brings to mind the touching custom prevailing up to our present time in placing the toys of a dead child upon its grave, a custom which has the same origin as that of the Indians, the acknowledgment of eternal life incorporated in matter and its transference into another world.

Page 109: "The widely prevailing custom of burying articles necessary for traveling with the dead to assist the spirits on their journey to their future abode, and to be of service to them during their residence there is begotten of their doctrine—the immortality of the soul. Some believe that as the things deposited in the graves decay, the spirits will take them away to be again united and used in the spirit-world, but the more general opinion is that the spirits being immaterial must use spiritual things, and they take, therefore, the articles and leave the matter behind."

Potentiation, then, is not a development of matter into force as has been supposed, not a kind of evolution from the homogeneous to the heterogeneous, but a disengaging of the medicinal qualities, forces, or properties inherent in all kinds of matter in its relation to the animal and human organism. The potencies which are developed by means of bringing an inert vehicle in contact with the crude substance under certain and necessary conditions must be considered to have been all residing in that crude substance from which they have been derived, or else it could not well be seen how the highest potencies in their action upon the organism could be similar to the action of the lower potencies and of the crude substance. The millionth centesimal potency of Sulphur acts just as well within its patho and hygiopoetic (metathetical) sphere as the crude substance or a third decimal potency, because in a sensitive person it produces just the same symptom of voluptuous itching with burning after scratching as is the immediate effect of brimstone taken in substance. It is, then, the homœopathic argument: like produces like, which proves the derivation of the potencies from the crude substance or all other things likewise composed by the minutules they consist of. By the most elaborate and ingenious test-instruments, contrivances, and processes of science we yet soon arrive at the fact that we cannot discern those minutules except in their aggregation, and though the limits of observation are constantly extending by the increase of knowledge and ingenuity of invention, yet we will never be able to show the existence and reality of the minutules by such means. The finest instruments given to us already are sensitive, intelligent, pure, and trusty persons who can give a good account of themselves in regard to observation of medicinal action upon themselves and upon whom the same can be observed objectively. For this reason the discovery of neural analysis by Jaeger is the first praiseworthy attempt on the part of the physiological school to use the human organism as a reagent upon those minutules which otherwise escape observation altogether. As Jaeger remarks very truly, there is hereafter no escape for the scientific man but to accept the facts furnished by this new method of investigation, neural analysis, and use them for the improvement of science and for the benefit of mankind.

It would, therefore, almost seem as if the question of potentiation altogether depends upon quantity, and so it does, for you may take the original drop or grain of the crude substance to contain as many minutules as you may please, more than any human brain can compute and think of, because there is ample room for them even in a grain or drop. Suppose you take it for granted to be so, then the object of potentiation would be to isolate the minutules from each other by the simplest means possible, and gain from the crude mass at least as many as you can, for it is impossible and unnecessary to gain them all for the purpose of healing.

How it is that high potencies act promptly and powerfully upon the human system in disease, and even in health depends upon observations and experiments made by men, who are able to judge in these matters. Besides having received the common medical education they must be also versed in the departments of physics and chemistry, and withal be thinkers able to follow the workings of their own minds—*i. e.*, they must be true philosophers—*i. e.*, lovers of wisdom. And not that alone, they must have thoroughly studied the works of the immortal Hahnemann and must have mastered the *Materia Medica Pura* as far as a human mind can master that vast storehouse of homœopathic facts. Then and only then can they do justice to this matter which has been held as a byword of foolishness. Surely our wisdom is foolishness if we are foolish enough to cast away what is offered in a simple and unpretending garb as wisdom. It is even so yet that the light shines in the darkness, and the darkness apprehended it not. They rather create man with its wonderful organism out of a primeval cell than to rise to the heavenly thought of a Creator who has made all things—even that cell—and holds them in His mighty grasp for evermore. But in regard to potentiation it is certainly true, as in all things, that God has laid His omnipotence into the minutule of our high potency.

*Ceterum censeo macrodosiam esse delendam.*

